# PREIMPLEMENTATION OF A QUALITY IMPROVEMENT PROGRAM FOR VETERANS LIVING IN COMMUNITY NURSING HOMES

**DOI:** 10.1093/geroni/igae098.0157

**Published:** 2024-12-31

**Authors:** Joan Carpenter, Ann Kutney-Lee, Andrew Murray, Mary Ersek

**Affiliations:** University of Maryland Baltimore, Baltimore, Maryland, United States; Corporal Michael J. Crescenz VA Medical Center, Philadelphia, Pennsylvania, United States; Corporal Michael J. Crescenz VA Medical Center, Philadelphia, Pennsylvania, United States; University of Pennsylvania, Philadelphia, Pennsylvania, United States

## Abstract

We designed Preferences Elicited and Respected for Seriously Ill Veterans through Enhanced Decision-Making (PERSIVED), a five-year VA-funded quality improvement program, to support VA clinicians with data and tools to ensure consistent documentation of life sustaining treatment (LST) preferences for Veterans who receive care in community nursing homes (CNHs). Guided by the VA Quality Enhancement Research Initiative (QUERI) Roadmap for Implementation and Quality Improvement, we designed the pre-implementation phase. Over 5 months, we conducted virtual meetings with partners at six VA CNH programs to: 1) engage site leadership and recruit champions; 2) assess barriers and facilitators to documenting LST preferences; 3) develop process maps of existing workflows; and 4) evaluate baseline knowledge, skills and resources related to these clinical practices. Through individual interviews (n=41) and 14 meetings with clinical and administrative partners, we recruited 19 champions. Common barriers included lack of knowledge/training and confusion about roles/responsibilities among VA CNH program clinicians. Facilitators included access to VA and CNH electronic medical records and positive relationships among VA and CNH staff. Across the programs in this sample, process maps revealed variability in formal systems for ensuring LST documentation in CNHs and VA. Using these findings, we tailored educational resources, developed site-specific audit tools for tracking VA and CNH LST documentation, and created processes that integrated LST documentation into existing VA CNH program workflows. Pre-implementation proved to be a critical step to ensure success and guided us in developing individualized implementation plans and addressing potential challenges to evidence-based practice adoption.

